# Coincidence of paroxysmal supraventricular tachycardia and panic disorder: two case reports

**DOI:** 10.1186/1744-859X-9-13

**Published:** 2010-04-12

**Authors:** Katharina Domschke, Paulus Kirchhof, Peter Zwanzger, Alexander L Gerlach, Günter Breithardt, Jürgen Deckert

**Affiliations:** 1 Department of Psychiatry, University of Münster, Münster, Germany; 2 Department of Cardiology and Angiology, and German Competence Network on Atrial Fibrillation (AFNET), University of Münster, Münster, Germany; 3 Institute of Psychology, University of Münster, Münster, Germany; 4 Department of Psychiatry, University of Würzburg, Würzburg, Germany

## Abstract

Panic disorder (PD) is characterised by sudden attacks of intense fear with somatic symptoms including palpitations and tachycardia. Reciprocally, palpitations caused by paroxysmal supraventricular tachycardia (PSVT) are commonly associated with anxiety and may therefore be misdiagnosed as PD. As demonstrated by two case reports, PSVT and PD can occur comorbidly in a chronological sequence, with PSVT possibly precipitating and maintaining PD via interoceptive processes or, alternatively, with PD increasing the risk for PSVT by elevating stress levels. As both PSVT and PD require different treatments, potentially helpful differential clinical diagnostic criteria are proposed.

## Background

Panic disorder (PD) is characterised by a lifetime prevalence of 1% to 3% and sudden attacks of intense fear accompanied by somatic, particularly cardiac symptoms such as palpitations, chest pain, and tachycardia: 89% of patients with PD complain of palpitations, with up to 25% of patients initially referred to cardiac clinics with atypical chest pain or palpitations being later diagnosed with PD [1]. Reciprocally, palpitations caused by paroxysmal supraventricular tachycardia (PSVT) are associated with anxiety in approximately 20% of patients and may therefore be misdiagnosed as PD [2-4]. In patients with PSVT, radiofrequency ablation offers a curative therapy and can reduce anxiety symptoms dramatically. After successful catheter ablation, a minority of patients has been reported to still suffer from panic symptoms, pointing to a possible true comorbidity in at least 4% of cases [5].

Based on two case reports of patients with comorbid PSVT and PD, neuropsychophysiological processes potentially driving this comorbidity will be discussed. Additionally, as both PSVT and PD require different treatments, potentially helpful differential clinical criteria will be proposed.

## Case presentations

### Patient 1

A 34-year-old female patient presented to the department of psychiatry in 2002 with a history of panic attacks since the age of 18, occurring two to three times per week lasting for about 10 to 30 min and presenting with somatic symptoms including palpitations and tachycardia not terminable by vagal manoeuvres, as well as feelings of derealisation/depersonalisation and fear of losing control, going crazy or dying. In 1996, the patient additionally began to suffer from palpitations diagnosed as PSVT with a sudden onset and duration of 6 h, terminable by vagal manoeuvres. PSVT attacks were not accompanied by the other panic-related symptoms described above and the patient could clearly differentiate between PSVT and a tachycardic state within a panic attack. During an invasive electrophysiological study in July 2000, a rapid typical AV nodal re-entrant tachycardia was diagnosed, with induced cycle lengths of 280 to 330 ms (corresponding to heart rates during the tachycardia of 180 to 220 bpm). Using radiofrequency catheter ablation, the slow pathway of the AV node was successfully ablated. Thereafter, PSVT attacks subsided while the patient continued to suffer from increasingly severe panic attacks corresponding to a pathological Hamilton Anxiety Scale (HAMA) score of 36 and an increased score of 33 on the Anxiety Sensitivity Index (ASI). Increased sensitivity to cardiac sensations was reflected by an elevated Body Sensation Questionnaire (BSQ) mean score of 2.35 at the time of referral in 2002. Antidepressive pharmacological treatment with mirtazapine (15 to 30 mg) was administered for 6 months. Additionally, the patient underwent cognitive-behavioural psychotherapy (CBT) (20 sessions) including psychoeducation about the role of interoceptive cues within the vicious circle leading to panic attacks as well as interoceptive exposure (compare with [6]). After therapy, the patient was completely free of panic-related symptoms as reflected by a HAMA score of 0.

### Patient 2

A 37-year-old woman was referred to the department of psychiatry in 2003 with panic attacks since the age of 23, which were aggravated by stressful life events and lasted between 15 to 30 min with typical symptoms as described above including palpitations not terminable by vagal manoeuvre. In addition, the patient reported palpitations since the age of 16, starting with two episodes per year and culminating in four episodes per week with a duration of 20 s up to 10 min diagnosed as PSVT in the department of cardiology. PSVT episodes were accompanied by anxiety, but were terminable by a vagal manoeuvre, did not imply feelings of derealisation/depersonalisation or fear of losing control, going crazy or dying and were clearly distinguishable from the 'newer' panic attacks. In November 2003, the patient was successfully ablated using radiofrequency current (compare to patient 1) that terminated PSVT symptoms. However, panic attacks continued to occur with an even increased frequency of about 1 per day, which corresponded to a HAMA score of 39, an increased ASI score of 40. Again, an elevated mean BSQ score of 2.53 in December 2003 mirrored increased interoceptive sensitivity particularly towards cardiac activity. After cognitive-behavioural psychotherapy (25 sessions) including interoceptive exposure as described above the patient was completely symptom free after 12 months of treatment and also at a long-term follow-up (November 2007: HAMA: 1).

## Conclusions

The present cases demonstrate that PSVT and PD can co-occur as two different diagnostic entities. Both patients could clearly differentiate between PD and PSVT symptoms: Differential diagnostic clues were that in contrast to panic attacks PSVT attacks were not accompanied by symptoms such as feelings of derealisation/depersonalisation or a fear of losing control, going crazy or dying. Conversely, panic attacks were not terminable by vagal manoeuvres. The possibility of true comorbidity of PSVT and panic disorder thus necessitates reciprocal inclusion of both nosological entities in the diagnostic evaluation of the respective diseases.

Given the chronological sequence of onset of the two comorbid diseases in the present cases, two different neuropsychophysiological interactions of PSVT symptoms and specific PD symptoms may be postulated.

Firstly, as in patient 1, PD might trigger PSVT as has previously been shown for ventricular premature beats or arrhythmias possibly via chronic mental and somatic stress [7].

Secondly, as in patient 2 and also in patient 1 after ablation, PSVT might initiate or aggravate anxiety states as an internal cue of panic and anxiety. This latter hypothesis is in line with anxiety disorder patients exhibiting increased interoceptive sensitivity, particularly to heart beat [8,9], as mirrored by elevated BSQ mean scores in both cases presented here. Also, elevated ASI scores as in both cases here have been shown to be associated with more accurate heart rate estimation suggesting an interaction of physiological arousal and state anxiety in interoceptive accuracy [10,11]. In anxiety disorders, not only has increased self-report of somatic sensations been observed, but also a subsequent dysfunctional cognitive appraisal of these sensations with a significant bias towards a danger-related and catastrophising interpretational style (for example, [12,13]). Consistently, self-rated fears of specific physical and psychological symptoms in PD patients as measured by the BSQ are primarily related to the catastrophising idea of having a heart attack [14]. Thus, increased cardiac awareness as precipitated by PSVT and further influenced by increased anxiety sensitivity may be a factor for the development and maintenance of panic disorder.

Assuming a reciprocal neuropsychophysiological interaction between PSVT and PD via either chronic mental and somatic stress or interoceptive processes, immediate and focused differential treatment such as radiofrequency ablation in PSVT or cognitive behavioural psychotherapy, if necessary in combination with pharmacological treatment (for example, with serotonin reuptake inhibitors (SSRI) or noradrenaline and selective serotonin agonists (NaSSA)) in PD is warranted in order to possibly avoid the precipitation of the respective other disorder.

## Competing interests

The authors declare that they have no competing interests.

## Authors' contributions

KD contributed to the acquisition and interpretation of data (clinical history and ratings) and conceived and designed the case report. JD initiated the case report after treating the patients in a psychiatric/psychotherapeutic setting. PK performed radiofrequency catheter ablation in both patients, AG was involved in psychotherapeutic interventions and psychometric description; both together with GB and PZ contributed to the manuscript with important intellectual content. All authors contributed to drafting the manuscript and gave approval to the final version.

## Consent

Written informed consent was obtained from the patients for publication of this case report.
